# A SINE-like insertion in intron 13 of the *ATP7A* gene is associated with a mild form of Menkes-like disease in a Cavalier King Charles Spaniel

**DOI:** 10.1093/jvimsj/aalag200

**Published:** 2026-09-02

**Authors:** Emily A Fulton, Cleo Schwarz, Vidhya Jagannathan, Tosso Leeb, Rodrigo Gutierrez-Quintana

**Affiliations:** School of Biodiversity, One Health and Veterinary Medicine, University of Glasgow, Glasgow, G61 1QH, United Kingdom; Chestergate Veterinary Hospital, Chester, CH1 6LT, United Kingdom; Institute of Genetics, Vetsuisse Faculty, University of Bern, 3001 Bern, Switzerland; Institute of Genetics, Vetsuisse Faculty, University of Bern, 3001 Bern, Switzerland; Institute of Genetics, Vetsuisse Faculty, University of Bern, 3001 Bern, Switzerland; School of Biodiversity, One Health and Veterinary Medicine, University of Glasgow, Glasgow, G61 1QH, United Kingdom

**Keywords:** *ATP7A*, *Canis lupus familiaris*, copper metabolism, Menkes disease, whole genome sequencing

## Abstract

A 7-month-old intact male Cavalier King Charles Spaniel was presented for persistent glucosuria despite normoglycemia, failure to thrive, chronic diarrhea, and cerebellar ataxia. Fanconi syndrome was diagnosed, but the neurologic abnormalities were not fully explained. As a consequence of the early onset Fanconi syndrome, a hereditary process was suspected. Whole genome sequencing identified a private hemizygous SINE-like insertion into the *ATP7A* gene, at the end of intron 13, near the start of exon 14*.* In humans, variants in *ATP7A* are associated with Menkes disease, a disorder of copper metabolism associated with a spectrum of clinical signs including progressive neurodegeneration and connective tissue abnormalities. Clinically affected dogs with variants in *ATP7A* have not been reported previously. Although this case appears to represent a mild phenotypic presentation of Menkes-like disease, it raises the possibility that copper disorders aside from copper-associated hepatitis might exist in dogs. Further genetic screening and phenotypic characterization of rare genetic variants associated with copper metabolism would be beneficial to expand our knowledge of copper disorders in dogs and allow potential early intervention and modeling for metabolic diseases in humans.

## Case description

A 7-month-old intact male Cavalier King Charles Spaniel initially presented to the referring veterinarian for hematuria. The dog was treated with a week course of amoxicillin-clavulanate (20 mg/kg PO q12h) for a presumptive urinary tract infection. Urinalysis identified glucosuria that persisted despite antimicrobial treatment. The dog was consistently normoglycemic despite the glucosuria and therefore was referred for further investigations. Additional history provided by the owner at the time of referral indicated that the dog had struggled to gain weight and thrive and had experienced chronic intermittent diarrhea. The owner also reported an abnormal gait, with intermittent loss of control of the pelvic limbs.

Clinical examination was unremarkable aside from a coarse hair coat. Neurologic examination identified moderate cerebellar ataxia, characterized by hypermetria affecting mainly the pelvic limbs, and inconsistent delayed postural reactions on the right pelvic limb. Mild cervical pain was detected on palpation. The remainder of the neurologic examination was unremarkable. Given the presence of normoglycemic glucosuria, medical evaluation was prioritized.

Complete blood cell count and serum biochemistry identified only mild increases in blood urea nitrogen (24.6 mg/dL; reference interval [RI], 7.0-23.8) and cholesterol (330 mg/dL; RI, 77-271) concentrations neither of which were deemed clinically relevant. Blood glucose concentration was in normal range (86 mg/dL; RI, 54-99). Given the history of poor growth, a bile acid stimulation test was performed, and results were normal (pre, 0.1 μmol/L; RI, 0.10; post, 0.2 μmol/L; RI, 0-15). An adrenocorticotropic hormone stimulation test excluded hypoadrenocorticism (plasma cortisol concentration pre, 3.7 μg/dL and post, 13.4 μg/dL). Serum cobalamin concentration was mildly decreased (268 pg/mL; RI, > 271 pg/mL).

Urinalysis identified persistent marked glucosuria, and urine culture was negative. Abdominal ultrasonography was unremarkable. Leptospirosis PCR on the urine was negative whereas serology also was not supportive of this differential diagnosis. Given the suspicion of Fanconi syndrome, venous blood gas analysis was performed and indicated normal pH of 7.35 with mildly decreased bicarbonate concentration (16.1 mEq/L; RI: 20.0-29.0). Urine amino acid profiling also was performed and was abnormal showing marked urinary loss of multiple amino acids including lysine, threonine, glutamine, citrulline, and cystine. Given the abnormal neurologic examination findings, urine organic acids also were analyzed and identified a mild increase in methylmalonate.

After investigations, the dog was diagnosed with Fanconi syndrome and suspected chronic inflammatory enteropathy. The dog was transitioned to a hydrolyzed diet (Purina HA) and cobalamin supplementation was started (Cobalaplex, Protexin), together with daily multivitamin and weekly amino acid supplementation.

The patient was regularly monitored and, despite showing a mild decrease in bicarbonate concentration, blood pH remained within normal limits. Several months after diagnosis, the dog developed persistent hypokalemia and was started on potassium supplementation (Kaminox, VetPlus).

Approximately 1 year after initial presentation, the patient remained clinically stable with respect to the Fanconi syndrome. However, progressive cerebellar ataxia and pain when jumping down from the sofa were reported. Neurologic examination identified a persistently hypermetric gait and pain on lumbosacral palpation. Orthopedic examination documented repeatable pain on manipulation of the elbows and possible reluctance to extend the left hip. Magnetic resonance imaging of the brain and vertebral column showed changes compatible with Chiari-like malformation without syrinx formation. Cerebrospinal fluid analysis was unremarkable. Radiographs of the elbows and coxofemoral joints were normal.

Given the early onset of Fanconi syndrome and the unexplained neurologic findings, a hereditary cause was suspected. Genomic DNA was isolated from EDTA blood of the affected dog, using a Maxwell RSC Whole Blood DNA Kit on a Maxwell RSC48 instrument according to the manufacturer’s protocol (Promega, Dübendorf, Switzerland). An Illumina TruSeq PCR-free DNA library with approximately 400 base pair (bp) insert size was prepared and sequenced at 19.6 times coverage on a NovaSeq 6000 instrument. The reads were mapped to the UU_Cfam_GSD_1.0 reference genome assembly as described previously.^1^ Filtering for private variants by comparing the sequence data of the affected dog against 1749 dogs of different breeds, excluding 8 same-breed dogs*,* identified 4 homozygous and 30 heterozygous protein-changing variants absent from all control genomes. None of these variants was in a gene previously associated with Fanconi syndrome or slowly progressive ataxia (Tables S1 and S2). We subsequently selected 6 functional candidate genes based on known metabolic pathways that could bridge neurologic dysfunction with renal tubular dysfunction, including candidate genes for Fanconi syndrome (*ALDOB*, *ATP7A*, *ATP7B*, *CTNS*, *ALDOB*, *GLUT2*, *OCRL1*). We visually investigated the short read alignments in the region of these genes in the Integrative Genomics Viewer (IGV) to search for structural variants that would not have been detected in our initial automated analysis of small variants, which identified a potential structural variant in a functional candidate gene. This approach led to identification of a hemizygous insertion in the X-chromosomal *ATP7A* gene, encoding ATPase copper transporting α. The insertion was at the end of intron 13, close to the boundary of exon 14 and can be formally designated as NC_049260.1:g.60,446,488_60,446,489insN[223] or NC_049260.1(XM_038587767.1):c.2404-3_-2insN[223]. It included a duplication of 16 nucleotides flanking the insertion site (Figure S1, File S1). The insertion was absent in whole genome sequencing data of 7 control dogs of the same breed.

To assess the functional effect of the insertion on transcription, we performed a semi-quantitative RT-PCR experiment. Total RNA was isolated from whole blood collected into PAXgene Blood RNA tubes of the affected dog and an unaffected male control dog using the PAXgene Blood RNA kit (PreAnalytiX, Hombrechtikon, Switzerland). Residual genomic DNA was removed using the QuantiTect Reverse Transcription Kit (Qiagen), and cDNA was synthesized. For RT-PCR, the forward primer 5′-TTTATTGTGTGTACCTGTACAGTTTTT-3′, spanning the exon boundaries of exon 12 and 13, and a reverse primer 5′-TCCCCTGTGATGAGGGACT-3′ spanning the exon boundaries of exon 15 and 16, were used. The RT-PCR products were visualized using a 5200 Fragment Analyzer instrument (Agilent, Basel, Switzerland) and sequenced on an ABI3730 DNA analyzer (Thermo Fischer Scientific, Waltham, MA, USA). The generated data were analyzed using the Sequencher 5.1 software (GeneCodes, Ann Arbor, MI, USA). The RT-PCR identified a complex pattern of aberrant splicing in the affected dog. Three distinct transcript species were identified and confirmed by Sanger Sequencing. The predominant splice product (388 bp; approximately 50% of the total transcript) was characterized by the complete skipping of exon 14, causing a frameshift and premature termination codon. A second aberrant transcript (288 bp; approximately 40% abundance) had a deletion consisting of parts of exon 13 and the entire exon 14, predicted to result in an in-frame deletion of 64 amino acids comprising the fifth transmembrane domain and parts of the third extracellular domain of the ATP7A protein, which is required for the dephosphorylation step of the catalytic cycle.^2^ Notably, the wild-type transcript was present only as a minor band (480 bp), accounting for approximately 10% of the identified transcripts. The control dog showed the presence of only one fragment (480 bp), consistent with the wild-type transcript (Figures S1 and S2).

The genetic findings prompted re-evaluation of the clinical presentation, because the genotype was consistent with Menkes disease, providing a more plausible explanation for the observed phenotype than the initially suspected Fanconi syndrome.

Plasma copper concentration was assessed in the patient and found to be low (7 μmol/L; RI, 7.5-16 μmol/L), whereas serum ceruloplasmin concentration was within the lower half of the RI (4.6 mg/dL; RI, 3.2-9.2 mg/dL).

## Discussion

In humans, 2 copper transport ATPases have been identified, *ATP7A* and *ATP7B*. Variants in *ATP7A* are associated with Menkes disease (MIM#309400), occipital horn syndrome (OHS); (MIM#304150) and X-linked distal hereditary motor neuronopathy; (MIM#300489), whereas variants in *ATP7B* are associated with Wilson’s disease (MIM#277900), which represents a clinically distinct disorder of copper metabolism.^3^^,^^4^ Both ATPases are responsible for the delivery of copper to copper-dependent enzymes within the trans-Golgi network. Under conditions of increased intracellular copper concentrations, *ATP7A* mediates cellular copper efflux into the systemic circulation, whereas *ATP7B* facilitates biliary copper excretion. Although *ATP7A* is expressed ubiquitously, *ATP7B* expression predominantly occurs in hepatocytes.^5^

Menkes disease in humans is an X-linked recessive disorder of copper metabolism caused by pathogenic variants in the *ATP7A* gene and therefore predominantly affects males.^6^ This disorder encompasses a spectrum of disease resulting from impaired ATP7A-mediated copper transport and is classically associated with progressive neurodegeneration, connective tissue abnormalities, and characteristic hair changes such as pili torti (“kinky hair”).^7^ Numerous *ATP7A* variants have been described, resulting in variable clinical phenotypes ranging from severe Menkes disease to milder allelic disorders such as OHS.^7^ Classic Menkes disease typically presents in early infancy with hypotonia, seizures, failure to thrive, autonomic dysfunction, connective tissue abnormalities, and characteristic facial features, with untreated individuals often succumbing in early childhood.^7^ In contrast, OHS represents a milder phenotype characterized primarily by connective tissue abnormalities, including joint laxity, bladder diverticula, and pathognomonic occipital exostoses. Between these 2 ends of the phenotypic spectrum lies a milder form of Menkes disease, in which affected individuals might demonstrate developmental delay, cerebellar ataxia, pili torti, and variable connective tissue abnormalities, but without seizures or early childhood mortality. In these individuals, the symptoms overlap between OHS and classic Menkes disease and they are referred to as “atypical” Menkes disease.^7^ The variability in clinical presentation likely reflects different degrees of residual ATP7A function associated with specific variants. Classic Menkes disease is associated with minimal functional ATP7A whereas in OHS and “atypical” Menkes disease some functional protein is present.^7^ With only approximately 10% of the wild-type transcript remaining, it is likely that the dog described herein experienced a deficit in systemic copper transport. The presence of only a small fraction of wild-type transcript suggests a hypomorphic phenotype. This feature might explain the dog’s survival past the neonatal period, presenting instead with a slightly milder, albeit progressive, clinical course compared with the complete null alleles typically associated with classic Menkes disease in humans.

The combination of cerebellar ataxia, failure to thrive, and coarse hair coat without severe early neurologic deterioration therefore might be most consistent with a milder Menkes-like phenotype within this spectrum.^7–9^

The primary defect in Menkes disease is impaired cellular copper transport. Within the gastrointestinal tract, defects in ATP7A prevent efficient export of absorbed copper into the bloodstream, resulting in low serum copper and ceruloplasmin concentrations. Defects in ATP7A also can lead to copper accumulation in tissues such as the kidneys and enterocytes, the exception occurs in the brain and liver in which copper deficiency is seen.^10^ The decreased copper concentrations in the brain reflect impaired ATP7A-mediated transport across the blood–brain and blood–cerebrospinal fluid barriers. Copper appears to become trapped within these barrier structures, resulting in copper deficiency in neurons and glial cells.^6^ In the liver, the main ATPase responsible for copper metabolism is ATP7B rather than ATP7A and hepatic copper deficiency is thought to be caused by trapping of copper within other tissues.

In contrast, Wilson’s disease arises from variants in *ATP7B*, that result in impaired biliary copper excretion and subsequent hepatic accumulation, which can lead to hepatocellular injury, chronic hepatitis, and ultimately cirrhosis. When hepatic storage capacity is exceeded, excess copper is released systemically and can deposit in the brain, resulting in neuronal degeneration and neurologic dysfunction.^11^

In veterinary literature, heritable disorders of copper metabolism in dogs classically manifest as copper accumulation. The main example is copper-associated hepatitis (CAH) reported in various dog breeds. In the Bedlington Terrier, an autosomal recessive disease caused by deletion of exon 2 of *COMMD1* is a well-recognized cause of CAH.^12^ In such dogs, hepatic copper concentrations 25 times higher than normal have been documented.^13^ Labrador Retrievers are also a breed classically associated with CAH, but unlike in Bedlington Terriers, this condition is not thought to be associated with *COMMD1* variants.^14^ Although the cause of CAH in Labrador Retrievers appears to be multifactorial, genetic studies have identified alterations in both the *ATP7B* and *ATP7A* genes within the breed. The *ATP7B*:p.Arg1453Gln substitution has been shown to be associated with copper accumulation, establishing the Labrador Retriever as a potential spontaneous animal model for Wilson’s disease in humans.^15^

Although naturally-occurring *ATP7A* variants have been reported in dogs, they have not previously been linked to a clinical phenotype resembling Menkes disease. A missense variant in *ATP7A* (NC_049260.1:g. 60422217C > T, XM_005641519.2:c.980C > T) identified in Labrador Retrievers was shown to modify hepatic copper concentrations, appearing to exert a protective effect against copper accumulation rather than causing systemic copper deficiency and was not associated with a clinically apparent copper deficiency phenotype.^15^ In vitro functional studies in canine fibroblasts identified altered cellular copper handling consistent with a partial decrease in ATP7A activity, but affected dogs did not exhibit neurologic or connective tissue abnormalities characteristic of Menkes disease in humans.^15^ To our knowledge, no spontaneous canine *ATP7A* variant has been previously reported to cause a clinically apparent copper deficiency syndrome analogous to Menkes disease in humans. Although our case did not exhibit the full spectrum of clinical signs characteristic of classic Menkes disease in humans, the presentation is consistent with a milder phenotype in the dog. The clinical presentation, specifically the neurologic and dermatologic abnormalities, and the genetic findings are further supported by the low serum copper and borderline low ceruloplasmin concentrations. Although typically decreased in Menkes disease, serum copper and ceruloplasmin concentrations might remain within or near the RI, particularly in milder phenotypes such as OHS, or in cases with residual ATP7A activity, as suspected in our case. Therefore, normal ceruloplasmin concentrations should not be used to exclude the diagnosis, especially when other clinical and molecular indicators of copper transport deficiency are present.

Glucosuria and proteinuria are not commonly reported in humans with Menkes disease, but renal copper accumulation has been well documented.^5^^,^^16^^,^^17^ Glucosuria was the initial presenting complaint in our case. We hypothesize that the glucosuria and mild proteinuria seen in this dog might have been caused by damage secondary to copper accumulation within the kidney. This hypothesis is supported by the fact that a small number of Labrador retrievers with CAH have been found to develop renal glucosuria and tubular dysfunction secondary to increased renal copper concentrations, with evidence of renal tubular vacuolization, degeneration, and regeneration observed on light microscopy.^18^ Furthermore, one report in the literature describes a child with Menkes disease who developed renal tubular dysfunction leading to electrolyte disturbances.^19^ The dog described in our case report had persistent hypokalemia, suggesting that urinary potassium wasting caused by tubular dysfunction secondary to copper accumulation might have been present.

Treatment of Menkes disease in humans is centered on providing early parenteral copper histidinate supplementation.^6^^,^^20^ Treatment for patients with OHS tends to be more focused on symptom control, and copper supplementation is not frequently provided although treatment trials in patients with OHS are sparse.^21^ The dog of our report was not supplemented with copper, but doing so might be beneficial in more severely affected animals.

We report a dog with a genetic variant in *ATP7A* and clinical signs suggestive of mild Menkes disease. This case raises the possibility that additional copper disorders in addition to CAH might exist in the dog. Although our dog appeared to have a mild phenotype, it is possible that dogs with a phenotype closer to classic Menkes disease might exist. Our report emphasizes the need for expanded genetic screening and phenotypic characterization of rare genetic variants associated with copper metabolism. Identification of variants in genes involved in copper metabolism might enable early intervention strategies and add to the knowledge of copper disorders in dogs, which would benefit our patients and potentially serve as models for metabolic disease in humans.

## Supplementary Material

Supplementary_material_aalag200

## Data Availability

All data in text or supplementary material. Genome public deposit.

## Off-label antimicrobial declaration

Authors declare no off-label use of antimicrobials.

## Institutional animal care and use committee or other approval declaration

All tests performed with owner consent as part of clinical practice.

## Abbreviations

OHS: occipital horn syndrome
CAH: copper associated hepatitis
