# Supplementary material for: A SINE-like insertion in intron 13 of the ATP7A gene is associated with a mild form of Menkes-like disease in a Cavalier King Charles Spaniel
Source: J Vet Intern Med. 2026 Sep 2;40(5):aalag200. doi: 10.1093/jvimsj/aalag200 (PMC13537520; doi:10.1093/jvimsj/aalag200)
Supplement: Supplementary file 1 [file supplementary_material_aalag200.zip › File_S1_SINE_insertion.docx]

**File S1. Sequence context of the 223 bp insertion into intron 13 of the canine *ATP7A* gene**

Genetic variant:

ChrX: NC_049260.1:g.60,446,488_60,446,489insN[223] (XM_038587767.1):c.2404-3_-2insN[223])

The sequence of UU_Cfam_GSD_1.0 assembly, chromosome X genomic sequence, positions 60,446,401 - 60,446,640 (NC_049260.1) is indicated below. The 92 nt sequence of *ATP7A* exon 14 (of 26 exons total) is given in black CAPITAL LETTERS. Lowercase letters indicate intronic bases.

The sequence of the 223 bp insertion is given in blue. The inserted sequence begins with a T-rich sequence that resembles the polyA-tail of a SINE insertion in reverse complementary orientation. However, the inserted sequence is largely composed of simple repeats and a genuine canine SINE is no longer recognizable.

16 nt duplicated sequences flanking the insertion site are underlined.

```
60,446,401      atgttaagta ttatatcttt attttaggta aaacctgagc tttttttttt tttgccttag    60,446,460
60,446,461      cattgttagc atgtttattt tcatttat                                       60,446,488
                             16 nt duplication flanking the insertion
60,446,488.1    tttttttttt aaattttttt tttaatttta tttatttatg atagtcacat agagagagag    60,446,488.60
60,446,488.61   agagagagag agagaggcag agacataggc agagggagaa gcaggctcca tgcaccggga    60,446,488.120
60,446,488.121  gcctgacgtg ggattcgatc cggggtttcc aggatcgcgc cctgggccaa aggcaggcgc    60,446,488.180
60,446,488.181  caaaccactg cgccatccag ggatcccgtt tattttcatt tat                      60,446,488.223
                                             16 nt duplication flanking the insertion

60,446,489                                    ag GGAAAAACAT CAGAGGCACT TGCCAAGCTA    60,446,520
60,446,521      ATTTCACTAC AAGCTACAGA AGCAACTATT GTAACTCTTG ACTCTGATAA TATTCTCCTA    60,446,580
60,446,581      AGgtatttat tttcattctt cccccttctc attttaactt cttatgaaaa atgtcgaata    60,446,640
```
