# Supplementary material for: A SINE-like insertion in intron 13 of the ATP7A gene is associated with a mild form of Menkes-like disease in a Cavalier King Charles Spaniel
Source: J Vet Intern Med. 2026 Sep 2;40(5):aalag200. doi: 10.1093/jvimsj/aalag200 (PMC13537520; doi:10.1093/jvimsj/aalag200)
Supplement: Supplementary file 1 [file supplementary_material_aalag200.zip › FigureS1_Details_of_the_ATP7A_Insertion_R1.pdf]

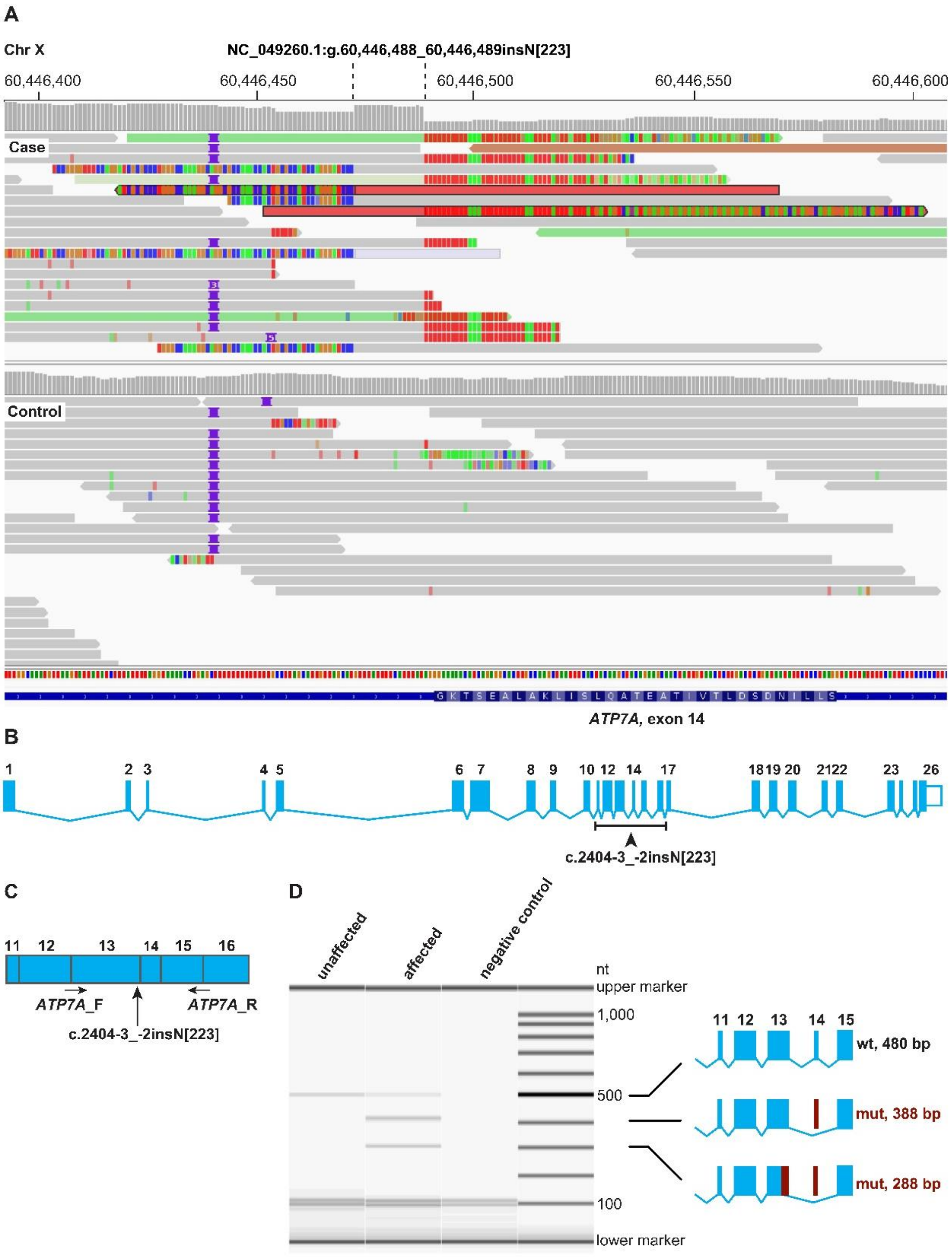

Figure S1. Details of the *ATP7A* insertion. (**A**) Integrative Genomics Viewer (IGV) screenshot illustrating the structural variant. Read alignments from the affected dog (top) are compared to an unaffected control (bottom). The affected dog shows an increased coverage over a 16 nt region spanning ChrX:60,446,473-60,446,488 (UU_Cfam_GSD_1.0). Multiple read alignments are soft-clipped at the boundaries of the duplicated 16 nt region. Colored reads indicate that their mates map to other chromosomes. These features are characteristic for an insertion of a repetitive element into the genome. (**B**) Schematic representation of the *ATP7A* transcript isoform X1 comprising 26 exons (XM_038587767.1). Exons are depicted as rectangles, introns as lines (not drawn to scale). The variant position is indicated. (**C**) Detail of cDNA showing exons 11-16. Position of primers for RT-PCR are indicated. Primers span exon boundaries to minimize amplification of unspecific genomic products. (**D**) RT-PCR using RNA isolated from whole blood. Experimental genotyping of the insertion by fragment size analysis on a capillary gel electrophoresis instrument. PCR amplification of the intron 13/exon 14 junction of the *ATP7A* gene was performed for the affected dog and a control dog. The displayed image is an electronically generated gel reconstruction (electropherogram). The unaffected animal shows only the expected wildtype band (480 bp). In the affected dog, the wildtype band is very weak and two additional bands; 388 bp and 288 bp, corresponding to a transcript lacking the entire exon 14 and a transcript lacking exon 14 and parts of exon 13 can be seen. The variant designation on the transcript level is thus r.[=, 2867_2958del, 2767_2958del]. The aberrant 388 bp cDNA band comprises roughly 50% and the 288 bp band roughly 40% of the total cDNA products in the affected dog. Lane 3 is the negative control (H_2O instead of cDNA added). The bands at the bottom of all three lanes with RT-PCR products correspond to molecules of < 100 bp in size and probably consist of residual unused primers, primer dimers or double-stranded primer aggregates with mismatches. Image contrast has been linearly enhanced across the entire panel to improve the visibility of the bands.
