# Supplementary material for: A SINE-like insertion in intron 13 of the ATP7A gene is associated with a mild form of Menkes-like disease in a Cavalier King Charles Spaniel
Source: J Vet Intern Med. 2026 Sep 2;40(5):aalag200. doi: 10.1093/jvimsj/aalag200 (PMC13537520; doi:10.1093/jvimsj/aalag200)
Supplement: Supplementary file 1 [file supplementary_material_aalag200.zip › FigureS2_R1.pdf]

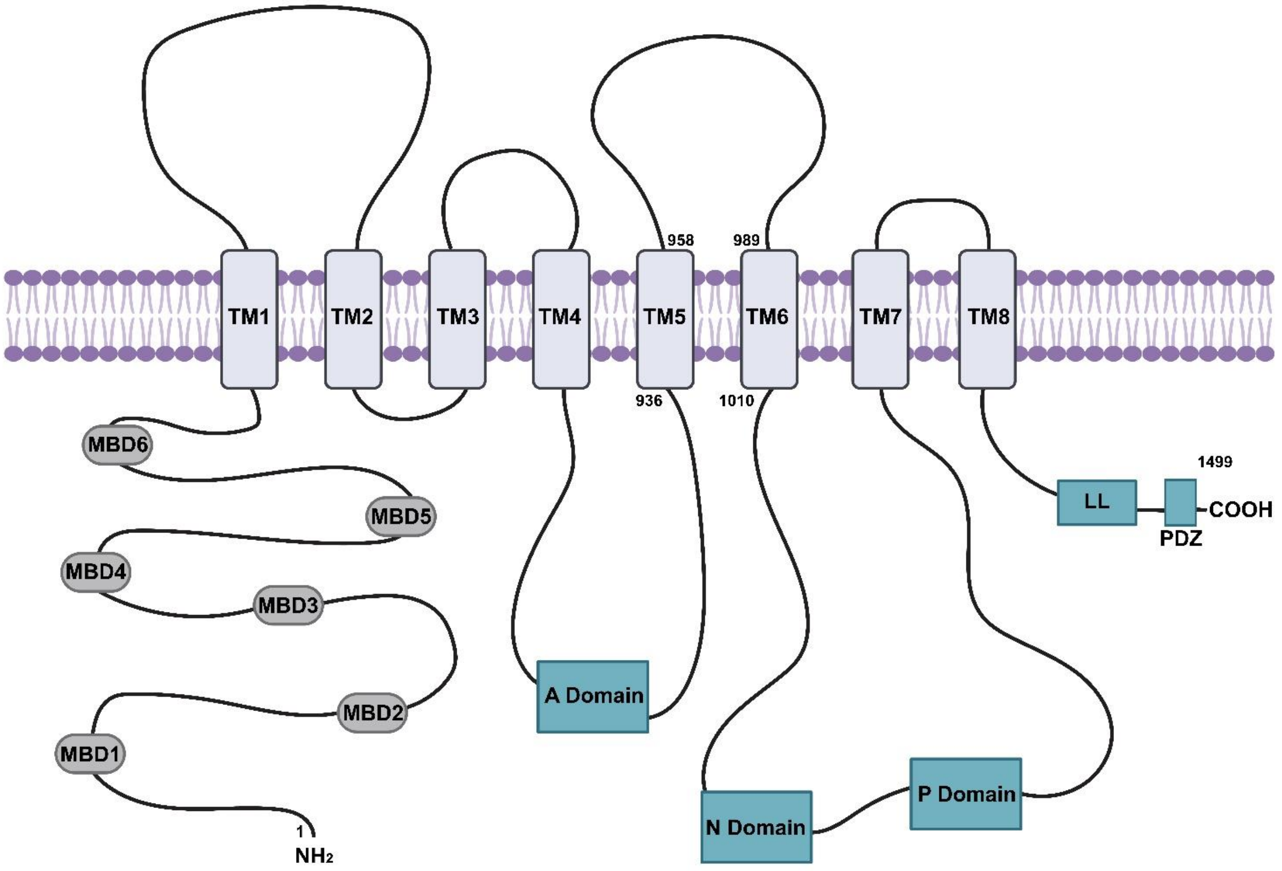

Figure S2. Schematic representation of the canine ATP7A protein (modified after Lenartowicz *et al.* 2015). The canine copper-transporting ATPase 1 isoform X1 (XP_038443695.1) is a 1499 amino acids protein with 8 transmembrane domains. Important domains are indicated. MBD = metal (Cu^{+}) binding domain, TM = transmembrane domain. A = actuator domain, N = nucleotide binding domain, P = phosphorylation domain, LL = di leucine motif, PDZ = PDZ-binding motif. Amino acid positions of selected functional domains are indicated.

The genomic insertion into intron 13 leads to expression of at least three different transcripts from the mutant allele. The predominant transcript (r.2867_2958del) comprises about 50% of all transcripts and contains a frameshift, XP_038443695.1:p.(I956Sfs*50). Thus the translation product from this transcript is predicted to lack TM domains 5-8 as well as other important functional domains.

The second-most abundant transcript (r.2767_2958del) comprises about 40% of all transcripts and contains an in-frame deletion that is predicted to remove 64 amino acids including TM5, XP_038443695.1:p.(Q923_F986del). It is thus not clear how a mutant protein might be integrated into the cell membrane. It seems very unlikely that the mutant protein could exert any relevant physiological function. (Created in BioRender. Schwarz, C. G. (2026) https://BioRender.com/30r3zr3)

Reference

Lenartowicz, M., Krzeptowski, W., Lipiński, P., Grzmil, P., Starzyński, R., Pierzchała, O., Møller, L.B. (2015) Mottled mice and non-mammalian models of Menkes disease. *Front. Mol. Neurosci.* 8:72. doi: 10.3389/fnmol.2015.00072.
